# Targeting tumor-derived NLRP3 reduces melanoma progression by limiting MDSCs expansion

**DOI:** 10.1073/pnas.2000915118

**Published:** 2021-03-01

**Authors:** Isak W. Tengesdal, Dinoop R. Menon, Douglas G. Osborne, Charles P. Neff, Nicholas E. Powers, Fabia Gamboni, Adolfo G. Mauro, Angelo D’Alessandro, Davide Stefanoni, Morkos A. Henen, Taylor S. Mills, Dennis M. De Graaf, Tania Azam, Beat Vogeli, Brent E. Palmer, Eric M. Pietras, James DeGregori, Aik-Choon Tan, Leo A. B. Joosten, Mayumi Fujita, Charles A. Dinarello, Carlo Marchetti

**Affiliations:** ^a^Department of Medicine, University of Colorado Denver, Aurora, CO 80045;; ^b^Department of Internal Medicine, Radboud Institute of Molecular Life Sciences, Radboud University Medical Center, 6525 GA Nijmegen, The Netherlands;; ^c^Department of Dermatology, University of Colorado Denver, Aurora, CO 80045;; ^d^Virginia Commonwealth University Pauley Heart Center, Virginia Commonwealth University, Richmond, VA 23230;; ^e^Department of Biochemistry and Molecular Genetics, University of Colorado Denver, Aurora, CO 80045;; ^f^Faculty of Pharmacy, Mansoura University, Mansoura 35516, Egypt;; ^g^Division of Hematology, Department of Medicine, University of Colorado Denver, Aurora, CO 80045;; ^h^Department of Biostatistics and Bioinformatics, Moffitt Cancer Center, Tampa, FL 33612

**Keywords:** NLRP3, IL-1, MDSCs, immunosuppression

## Abstract

Interleukin-1β (IL-1β)–mediated inflammation suppresses antitumor immunity, leading to the generation of a tumor-permissive environment, tumor growth, and progression. Here, we demonstrate that nucleotide-binding domain, leucine-rich containing family, pyrin domain-containing-3 (NLRP3) inflammasome activation in melanoma is linked to IL-1β production, inflammation, and immunosuppression. Analysis of cancer genome datasets (TCGA and GTEx) revealed greater NLRP3 and IL-1β expression in cutaneous melanoma samples (*n* = 469) compared to normal skin (*n* = 324), with a highly significant correlation between NLRP3 and IL-1β (*P* < 0.0001). We show the formation of the NLRP3 inflammasome in biopsies of metastatic melanoma using fluorescent resonance energy transfer analysis for NLRP3 and apoptosis-associated speck-like protein containing a CARD. In vivo, tumor-associated NLRP3/IL-1 signaling induced expansion of myeloid-derived suppressor cells (MDSCs), leading to reduced natural killer and CD8^+^ T cell activity concomitant with an increased presence of regulatory T (Treg) cells in the primary tumors. Either genetic or pharmacological inhibition of tumor-derived NLRP3 by dapansutrile (OLT1177) was sufficient to reduce MDSCs expansion and to enhance antitumor immunity, resulting in reduced tumor growth. Additionally, we observed that the combination of NLRP3 inhibition and anti–PD-1 treatment significantly increased the antitumor efficacy of the monotherapy by limiting MDSC-mediated T cell suppression and tumor progression. These data show that NLRP3 activation in melanoma cells is a protumor mechanism, which induces MDSCs expansion and immune evasion. We conclude that inhibition of NLRP3 can augment the efficacy of anti–PD-1 therapy.

## Significance

The nucleotide-binding domain, leucine-rich containing family, pyrin domain-containing-3 (NLRP3) inflammasome, an intracellular complex that regulates maturation and release of interleukin (IL)-1β, is active in biopsies of metastatic melanoma. Here, we demonstrate that NLRP3 activation in melanoma cells drives tumor progression in mice. Subsequent to NLRP3 activation in melanoma cells, IL-1β induces melanoma-associated inflammation, resulting in immunosuppression. Oral administration of a single NLRP3 inhibitor (OLT1177) reduces melanoma growth and melanoma-associated myeloid-derived suppressor cell expansion. Inhibition of the NLRP3 signaling in combination with anti–PD-1 revealed augmented efficacy compared to monotherapy. These data propose that NLRP3 is a therapeutic target for human melanoma.

Tumorigenesis is initiated by genomic alterations, leading to cell transformation, proliferation, and resistance to apoptotic signals, which ultimately lead to metastasis and tissue invasion. Tumor progression is also linked to dysregulated inflammation, which is characterized by cytokine signaling between cancer and noncancer cells (1, 2). The proinflammatory cytokine interleukin-1β (IL-1β) mediates several inflammatory diseases and is a pivotal cytokine in initiating inflammatory responses (3). In the context of malignancy, IL-1β is a validated target in mouse models of cancer, including melanoma, where the cytokine contributes to immunosuppression, angiogenesis, metastasis, and regulation of myeloid-derived suppressor cells (MDSCs) (1, 2, 4, 5). In humans, IL-1β is overexpressed in biopsies from metastatic melanoma patients, suggesting a possible role in the melanoma-induced inflammation (6).

Processing of IL-1β is largely governed by inflammasomes, cytosolic macromolecular complexes responsible for the conversion of biologically inactive IL-1β and IL-18 precursors into their active forms via caspase-1 cleavage (7). The nucleotide-binding domain, leucine-rich containing family, pyrin domain-containing-3 (NLRP3) is the most studied of the inflammasome sensors driving IL-1β–mediated conditions from sterile inflammation to rare hereditary syndromes (8, 9). NLRP3 is particularly relevant to the processing of IL-1β in melanoma because NLRP3 is constitutively expressed in melanoma cell lines (6) and NLRP3 polymorphisms are linked to increased risk to develop melanoma (10). Whereas these studies indicate a possible role for NLRP3 in melanoma progression, the biological function for NLRP3 in melanoma remains unclear. Furthermore, although it is well known that inflammation participates in the development and progression of melanoma (11–13), the inflammatory pathways that drive this process are still poorly characterized and no therapy developed to date is actually designed to specifically target inflammatory pathways in melanoma. Here, using genetic models of NLRP3 depletion and a specific pharmacological inhibitor of NLRP3 (14), we show that NLRP3 represents a melanoma intrinsic pathway exploited for tumor-mediated immune escape. We demonstrate that tumor-derived NLRP3 activation induces MDSC expansion, which suppresses recruitment and activation of antitumor immunity.

From a clinical standpoint, immune checkpoint therapy (ICT) has significantly improved the outcome for melanoma patients, and numerous studies have demonstrated that expression of PD-1/PD-L1 and CTLA4 are often predictors for efficacy of immunotherapy. However, the number of patients that are unresponsive to ICT or relapse continues to rise, and clinical data show that expression of immune checkpoints do not always correlate with responses (15). The limited response to monotherapy in some patients suggests that intrinsic pathways in melanoma cells, such as expression of checkpoint ligands, are not the only mechanisms that drive tumor progression. Identification of other tumor-specific strategies provides an opportunity to interrupt the oncogenic process and improve survival in this population. For example, melanoma-associated inflammation facilitates tumor progression (11, 16) and, specifically, is linked to IL-1β activity (4, 17). An approach for reducing IL-1β activity is via inhibition of NLRP3. Here, we show that disruption of the NLRP3 signaling in combination with ICT increases antitumor activity. The data support the concept that tumor NLRP3 activation represents an intrinsic pathway that favors tumor immune escape. Thus, targeting NLRP3 represents an innovative strategy for treating melanoma, especially in the context of immunotherapy resistance tumors.

## Results

### NLRP3 Inflammasome Activity Regulates IL-1β Secretion in Melanoma Cells.

**Fig. 1.**
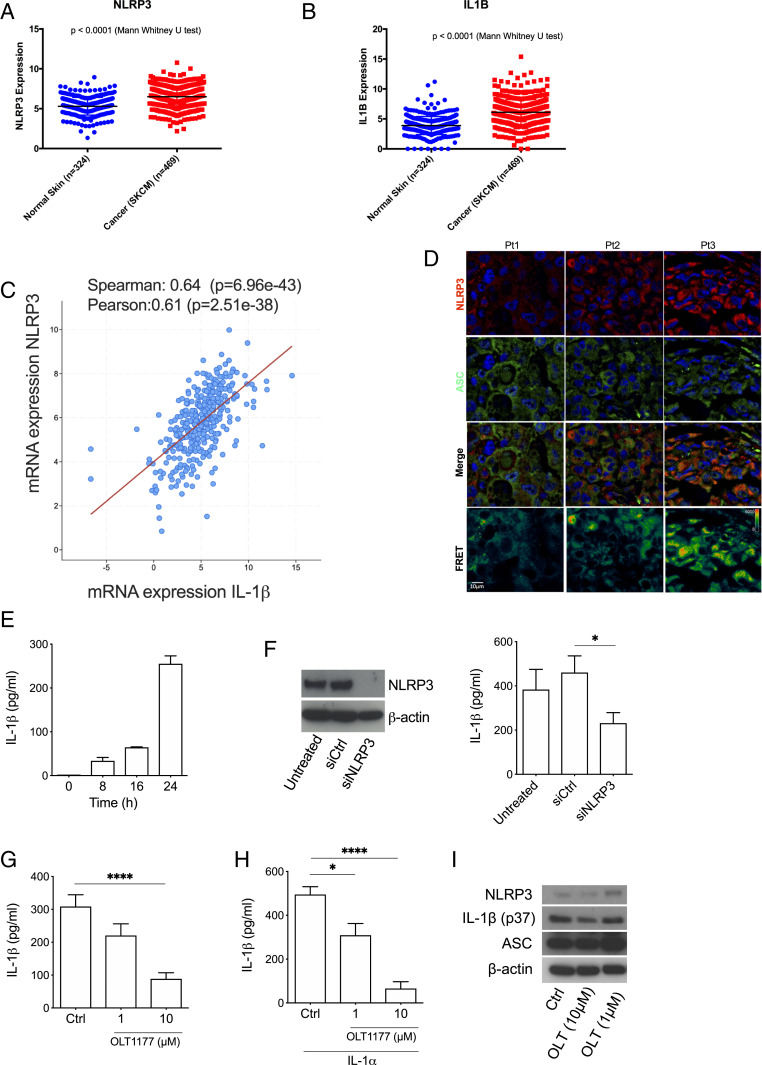
NLRP3 inflammasome mediates IL-1β secretion in human metastatic melanoma cells. (*A* and *B*) NLRP3 and IL-1β expression in human cutaneous melanoma (SKCM) and normal skin samples from GTEx and TCGA datasets. (*C*) Correlation between of IL-1β and NLRP3 expression in SKCM (*n* = 363). (*D*) Immunofluorescence stain and FRET for NLRP3 (red) and ASC (green) in metastatic melanoma biopsies. Each column represents a single patient. (*E*) Time course of spontaneous IL-1β secretion in 1205Lu cells (*n* = 3). (*F*) Representative Western blot analysis (*Left*) for NLRP3 at 24 h from 1205Lu cells (untreated) compared to cells transfected with scrambled (siCtrl) and NLRP3 (siNLRP3) siRNA and associated IL-1β release levels (*Right*) (*n* = 3). (*G*) Spontaneous IL-1β secretion in 1205Lu cells in presence of OLT1177 after 24 h (*n* = 4). (*H*) IL-1α-induced IL-1β secretion from 1205Lu cells in the presence of OLT1177 after 24 h (*n* = 4). (*I*) NLRP3, IL-1β (p37) and ASC intracellular expression from 1205Lu cells described in *G*. Data are expressed as mean ± SEM ****P < 0.0001, *P < 0.05.

Analyses of the Cancer Genome Atlas (TCGA) and the GTEx datasets exhibited highly significant increases in NLRP3 and IL-1β expression in cutaneous melanoma samples compared to normal skin (Fig. 1 *A* and *B*). NLRP3 expression correlated with the mRNA levels of IL-1β (*P* = 2.51e^−38^) (Fig. 1*C*). To determine whether NLRP3 expression associates with inflammasome formation, human melanoma skin biopsies from patients with metastatic melanoma were subjected to fluorescence resonance energy transfer (FRET) analysis for NLRP3 and ASC (apoptosis-associated speck-like protein containing a CARD), the inflammasome adaptor protein. FRET analysis revealed formed NLRP3 inflammasome in each of the samples (Fig. 1*D* and *SI Appendix*, Fig. S1). These data demonstrate increased NLRP3 expression and active inflammasome formation in melanoma biopsies and suggest that NLRP3 inflammasome activation contributes to the maturation of IL-1β in melanoma.

To determine the role of NLRP3 in IL-1β processing in melanoma, we specifically targeted NLRP3 in human melanoma cell lines. Cultured human metastatic melanoma 1205Lu cells exhibit a time-dependent spontaneous release of IL-1β (Fig. 1*E*), which was reduced with NLRP3 silencing using small-interfering RNA (siRNA) (Fig. 1*F* and *SI Appendix*, Fig. S2*A*). We next targeted NLRP3 with OLT1177, a small synthetic, orally active specific NLRP3 inhibitor (14) (*K*_D_ = 1.2 µM) (*SI Appendix*, Fig. S2*B*). OLT1177 blocks the ATPase activity of NLRP3, a function required for the formation of the NLRP3 inflammasome (14, 18). Treatment with OLT1177 reduced both spontaneous and IL-1α–induced secretion of IL-1β in 1205Lu cells (−73% and −87%, respectively) (Fig. 1 *G* and *H*). Expectedly, in 1205Lu cells, treatment with OLT1177 had no effect on NLRP3, ASC, and the IL-1β precursor (p37) protein levels when compared to the vehicle-treated cultures (Fig. 1*I*). The role of NLRP3 in processing IL-1β in melanoma cells was confirmed using A375 and HS294T cells, two different metastatic melanoma cell lines, as shown in *SI Appendix*, Fig. S2 *C*–*F*. No effect of OLT1177 on proliferation was measured in 1205Lu, A375, and HS294T (*SI Appendix*, Fig. S2*G*). These in vitro findings demonstrate constitutive NLRP3 inflammasome activation in human melanoma cells and its role in the processing and secretion of IL-1β.

### Inhibition of Tumor-Derived NLRP3 Reduces Melanoma Progression.

**Fig. 2.**
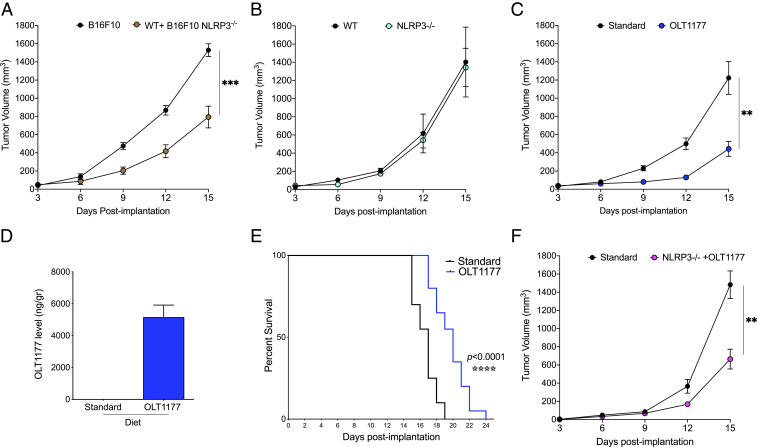
Inhibition of tumor-NLRP3 reduces melanoma progression. (*A*) Tumor growth in wild-type mice with B16F10 and B16F10 *nlrp3*^*−/−*^ (*n* = 5 per group). (*B*) Tumor growth in wild-type and *nlrp3*^*−/−*^ mice (*n* = 10 per group). (*C*) Tumor growth in mice fed standard or OL1177 diet (*n* = 15 per group). (*D*) Concentration of OLT1177 in primary tumors of mice fed standard and OLT1177 diet (*n* = 10). (*E*) Survival curve in mice fed standard or OLT1177 diet (*n* = 10 per group). (*F*) Tumor growth in wild-type mice fed standard diet and in *nlrp3*^*−/−*^ mice fed OLT1177 diet (*n* = 5 per group). ****P < 0.0001, ***P < 0.001, **P < 0.01.

Next, we evaluated the effect of NLRP3 inhibition in vivo. Considering the constitutive expression of NLRP3 in melanoma cell lines, mice were implanted subcutaneously with the murine melanoma cells line B16F10 and compared to mice injected with B16F10 cells where NLRP3 was deleted (B16F10 *nlrp3*^*−/−*^) (*SI Appendix*, Fig. S3*A*). As shown in Fig. 2*A*, NLRP3-deficient tumors exhibit significantly lower tumor growth compared to growth of native B16F10 cells. In order to determine the contribution of host-NLRP3 inflammasome on tumor progression, we also examined the growth of native B16F10 cells implanted in NLRP3-deficient mice (*nlrp3*^*−/−*^). Tumor growth was nearly the same in NLRP3-deficient mice as in WT mice (Fig. 2*B*).

Next, we evaluated the effect of blocking NLRP3 in vivo using OLT1177. Mice were implanted with native B16F10 melanoma cells, as described above, and fed an OLT1177-enriched diet or fed standard chow from the day of tumor implantation. Tumor-bearing mice fed OLT1177 exhibited a 65% reduction (*P* < 0.01) in tumor growth compared to mice fed standard chow (Fig. 2*C*). The reduction in tumor growth in mice with the OLT1177 diet was comparable to that achieved with daily intraperitoneal administration of anakinra, the recombinant form of the naturally occurring IL-1 receptor antagonist (IL-1Ra) (*SI Appendix*, Fig. S3*B*). Considering the observation that tumor-derived NLRP3 drives tumor progression (Fig. 2 *A* and *B*), we measured the concentration of OLT1177 in the tumors. As shown in Fig. 2*D*, the level of OLT1177 in the tumors reached concentrations sufficient to prevent the NLRP3 inflammasome formation, as reported in previous investigations (14, 19). In addition, we determined whether the reduction in tumor volumes with OLT1177 increased survival of the mice. As shown in Fig. 2*E*, increased survival was observed in mice fed OLT1177 compared to mice fed standard diet (*P* < 0.0001).

To further confirm that the role of NLRP3 in tumor progression is not of host origin but rather of tumor origin, we fed *nlrp3*^*−/−*^ mice with the OLT1177-enriched diet. As depicted in Fig. 2*F*, the feeding of OLT1177 to the *nlrp3*^*−/−*^ mice significantly reduced tumor growth (−58%; *P* < 0.01) compared to mice fed the standard diet.

The reduction in tumor growth in wild-type mice fed OLT1177 was also observed using implanted YUMM melanoma cells (*SI Appendix*, Fig. S3*C*); thus, specific targeting of NLRP3 is not limited to B16F10 cells. Overall, we conclude that NLRP3 of melanoma origin functions as an intrinsic signal for tumor progression.

### Tumor-Derived NLRP3 Drives Inflammation in Melanoma.

**Fig. 3.**
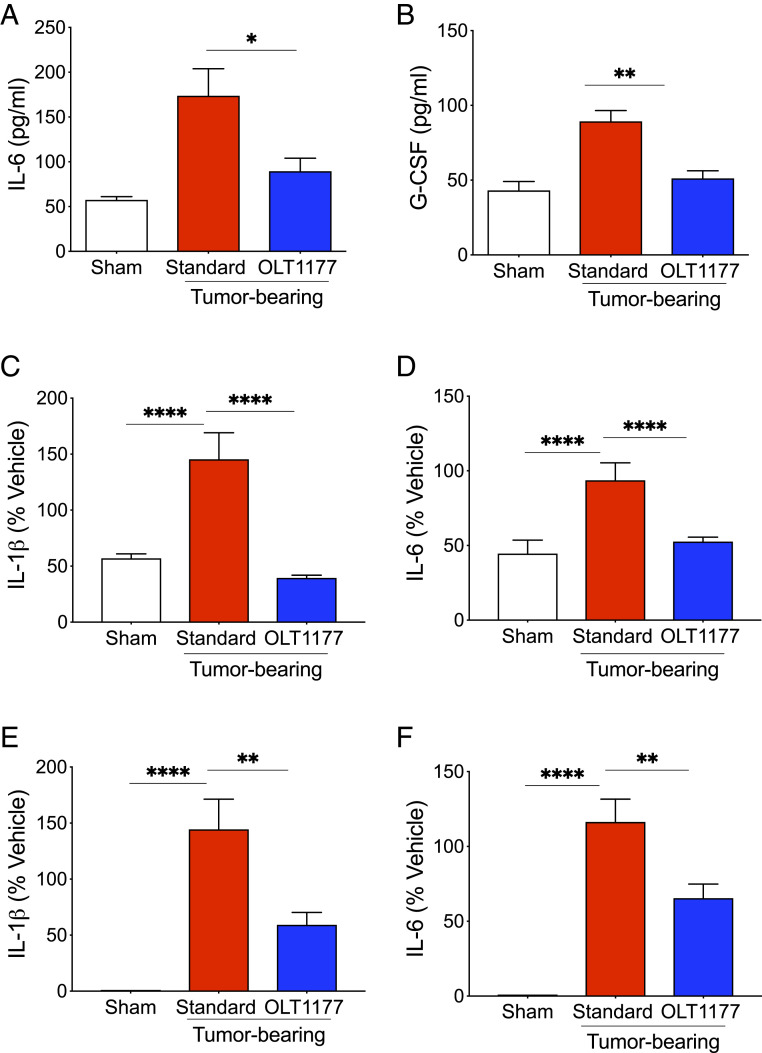
Tumor-NLRP3 activity drives melanoma-associated inflammation. (*A* and *B*) Mean ± SEM of plasma IL-6 (*A*) and G-CSF (*B*) in sham and tumor-bearing mice fed standard or OLT1177 diets (*n* = 4 to 6 per group). (*C*–*F*) Spontaneous IL-1β and IL-6 secretion in bone marrow (*C* and *D*) and spleen (*E* and *F*) -derived cell from sham and tumor-bearing mice fed standard or OLT1177 diets after 48 h of culture (*n* = 5). (*C*–*F*) Data are presented as percent vehicle with mean vehicle IL-1β levels of 135 pg/mL (*C*) and 53 pg/mL (*E*), and mean IL-6 levels of 1,396 pg/mL (*D*) and 890 pg/mL (*F*). ***P < 0.001, **P < 0,01, *P < 0.05.

We next determined whether NLRP3 inhibition with OLT1177 reduced systemic inflammation, a known tumor-promoting factor in human melanoma. We measured plasma levels of IL-6 and granulocyte-colony stimulating factor (G-CSF), as both are directly induced by IL-1β and are poor predictors of outcomes in melanoma (20–23). Tumor-bearing mice fed standard diet exhibited significantly higher plasma levels of IL-6 and G-CSF compared to nontumor-bearing mice (Fig. 3 *A* and *B*). In mice fed the OLT1177 diet, IL-6 and G-CSF levels were significantly lower compared to the mice fed standard chow (*P* < 0.05 and *P* < 0.01, respectively) (Fig. 3 *A* and *B*). We next measured spontaneous production of IL-6 and IL-1β in ex vivo cultured bone marrow and spleen cells to determine whether tumor growth influenced host inflammatory responses. As shown in Fig. 3 *C*–*F*, bone marrow and spleen cells derived from tumor-bearing mice spontaneously released higher levels of IL-1β and IL-6 during 48 h in culture, compared to nontumor-bearing mice. In the same tissue-derived cells from mice fed OLT1177, the production of IL-1β was reduced by 61% from the bone marrow (*P* < 0.0001) and 41% from the spleen cells (*P* < 0.01); IL-6 was reduced by 48% from the bone marrow and 35% from spleen cells (*P* < 0.0001 and *P* < 0.01, respectively) (Fig. 3 *C*–*F*). Overall, these data indicate that tumor-derived NLRP3 activation induces local host inflammatory cytokines, which fuel systemic inflammation.

### NLRP3 Inhibition by OLT1177 Reduces MDSCs Expansion.

**Fig. 4.**
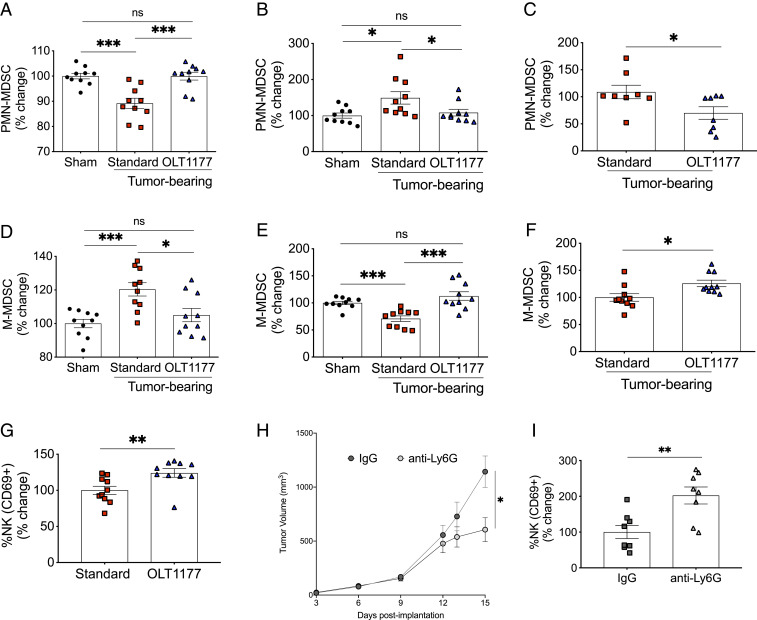
NLRP3 inhibition reduces myeloid-derived suppressor cells expansion. (*A*–*C*) Bone marrow (*A*), spleen (*B*), and lymph node (*C*) levels of PMN-MDSCs (CD11b^+^Ly6G^+^Ly6C^lo^) in nontumor-bearing mice (Sham) compared to tumor-bearing mice fed standard or OLT1177 diets. (*D*–*F*) Bone marrow (*D*), spleen (*E*), and lymph node (*F*) levels of M-MDSCs (CD11b^+^Ly6G^−^Ly6C^hi^) in Sham compared to tumor-bearing mice fed standard or OLT1177 diets. Data are presented as percent change of MDSCs in Sham set at 100. (*G*) CD69^+^ NK cell levels in primary tumors of mice fed standard or OLT1177 diet. (*H*) Tumor growth curve of B16F10 cells in mice receiving control IgG or anti-Ly6G (*n* = 10 per group). (*I*) CD69^+^ NK cell in primary tumors from mice receiving IgG or anti-Ly6G described in *H* (*n* = 8per group). ***P < 0.001, **P < 0.01, *P < 0.05.

Considering the agonist role of IL-1β on MDSCs (24–26) and the role of these cells in tumor-associated immunosuppression, we assessed the effect of NLRP3 inhibition on MDSCs. Cells isolated from the bone marrow, spleen, and lymph nodes were analyzed for the two primary MDSCs subtypes: Polymorphonuclear MDSCs (PMN-MDSC) expressing CD11b^+^Ly6G^+^Ly6C^lo^ and monocytic MDSCs (M-MDSC) expressing CD11b^+^Ly6G^−^Ly6C^hi^. As shown in Fig. 4*A*, bone marrow cells from tumor-bearing mice exhibited significantly reduced PMN-MDSCs compared to nontumor-bearing mice (*P* < 0.001). In the spleen, the level of PMN-MDSCs of tumor-bearing mice was increased compared to nontumor-bearing mice (Fig. 4*B*). However, in mice fed OLT1177 diet, we observed a restoration of the PMN-MDSC population to the level observed in the nontumor-bearing mice (Fig. 4 *A* and *B*). Analysis of the draining lymph nodes also revealed a reduction in PMN-MDSCs in mice treated with OLT1177 compared to standard food (Fig. 4*C*). As depicted in Fig. 4*D*, bone marrow M-MDSCs levels were increased in tumor-bearing mice fed the standard diet (*P* < 0.001) when compared to nontumor mice. As with PMN-MDSCs, OLT1177 normalizes the levels of these cells to those measured in nontumor-bearing mice (Fig. 4*D*). In the spleen and lymph nodes, M-MDSC were lower compared to the nontumor-bearing mice but normalized with the OLT1177 diet (Fig. 4 *E* and *F*). These data reveal that NLRP3 signaling influences MDSCs expansion and that NLRP3 inhibition is sufficient to normalize MDSC populations to those of nontumor-bearing mice.

Because PMN-MDSCs inhibit natural killer (NK) cell activity (24, 27), we assessed activation of NK cells in the primary tumors. We observed a significant increase in the number of NK cells expressing the early myeloid activation maker CD69 in primary tumors of mice fed OLT1177 diet compared to standard diet (*P* < 0.01) (Fig. 4*G*). Consistent with tumor-derived NLRP3 driving immunosuppression, *nlrp3*^*−/−*^ mice showed no change in activated NK cells in primary tumors (*SI Appendix*, Fig. S4*A*).

To demonstrate the relationship between MDSCs and tumor progression in this model, tumor growth and NK activation was measured following neutralization of MDSCs using an antibody against Ly6G. Administration of anti-Ly6G to melanoma-bearing mice resulted in reduced tumor growth with increased infiltration of NK cells when compared to an IgG isotype control antibody (Fig. 4 *H* and *I*). Other cell types of the myeloid lineage were also assessed to determine changes in early precursors of myeloid and lymphoid lineages. However, tumor-bearing mice fed OLT1177 did not show evidence of reprogrammed myelopoiesis in the bone marrow when compared to tumor-bearing mice fed the standard diet (*SI Appendix*, Fig. S4 *B* and *C*). These findings support the role of MDSCs in the immunosuppression of this model but that the changes observed in the MDSCs populations with inhibition of the NLRP3 inflammasome are not driven by changes in myelopoiesis.

### NLRP3 Blockade Increases Efficacy of Anti–PD-1.

**Fig. 5.**
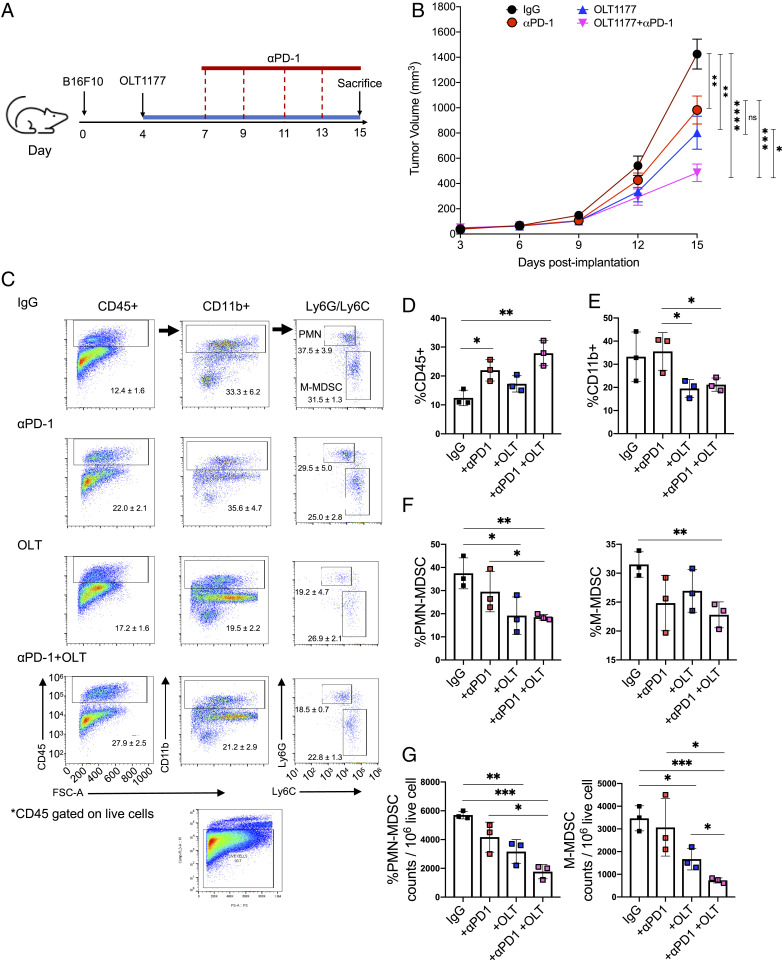
NLRP3 inhibition augments the therapeutic effect of anti-PD-1 treatment. (*A*). Schematic of the experimental design. (*B*) Tumor growth in mice following IgG, anti–PD-1, OLT1177 and the anti–PD-1 in combination with OLT1177 (*n* = 10 per group). (*C*) Gating strategy for CD45^+^, CD11b^+^, and Ly6G/Ly6C live cells in the TME from mice described in *A*. (*D*) Flow cytometry analysis of CD45^+^ cells in the TME. (*E*) Flow cytometry analysis of CD11b^+^ cells in the TME. (*F*) Flow cytometry analysis of PMN-MDSC (*Left*) and M-MDSC (*Right*) present in the TME. (*G*) Cell counts for PMN-MDSC (*Left*) and M-MDSC (*Right*) present in 10^6^ live tumor cells. Data expressed as mean ± SEM, ****P < 0.0001, ***P < 0.001, **P < 0.01, *P < 0.05.

With the reduction in MDSCs using NLRP3 inhibition, we sought to enhance the antitumor T cell response of anti–PD-1 by adding NLRP3 inhibition. The mice were originally placed on standard diet and implanted with B16F10 cells (experimental day 0). Four days after the implantation (experimental day 4), two groups initiated OLT1177 enriched food and two groups remained on the standard diet. Three days later (experimental day 7), mice on the standard or OLT1177 diet received anti–PD-1 antibody or a matching IgG control antibody intraperitoneally (Fig. 5*A*). As shown in Fig. 5*B*, anti–PD-1 and OLT1177 treatment each reduced tumor size by 32% (*P* < 0.01) and 44% (*P* < 0.01), respectively, compared to mice treated with the control IgG. Importantly, mice that received OLT1177 plus the checkpoint inhibitor showed a further reduction of 66% (*P* < 0.0001) when compared to mice treated with the IgG control (Fig. 5*B*). When compared to each of the monotherapies, the combination resulted in a further reduction in tumor volume of 51% compared to anti–PD-1 only (*P* < 0.001) and 40% compared to OLT1177 only (*P* < 0.05) (Fig. 5*B*).

Next, we measured the number of infiltrating cells in the tumor-microenvironment (TME) of mice treated with the above regimen. Fig. 5*C* illustrates the gating strategy. The TME of mice receiving anti–PD-1 or combination therapy with OLT1177 exhibited increased infiltration of CD45^+^ cells (*P* < 0.05 and *P* < 0.01, respectively), whereas mice on OLT1177 diet alone exhibited no significant change from mice fed standard food (Fig. 5*D*). Mice on the OLT1177 diet showed a decrease of CD11b^+^ cells with anti–PD-1 treatment (Fig. 5*E*) (*P* < 0.05). The MDSC populations in the TME were also assessed. PMN-MDSCs were significantly decreased in mice receiving the OLT1177 diet (*P* < 0.05) (Fig. 5 *F*, *Left*). Anti–PD-1 alone showed no difference from control IgG, whereas the addition of OLT1177 diet to anti–PD-1–treated mice contained significantly less PMN-MDSCs compared to anti–PD-1 alone (*P* < 0.05) (Fig. 5 *F* and *G*, *Left*). Monotherapies with OLT1177 or anti–PD-1, as well as the combination of the two, exhibited significant reduction in M-MDSC compared to the IgG control (Fig. 5 *F* and *G* *Right*). Overall, these findings support the concept that the NLRP3 inhibition added to anti–PD-1 has greater efficacy in reducing tumor growth compared to either agent as a monotherapy. The data reflect the reduction in infiltrating MDSCs, which appear to be mostly driven by NLRP3 inhibition rather than by anti–PD-1.

### NLRP3 Inhibition in Combination with Anti–PD-1 Increases CD8^+^ T Cell Tumor Infiltration.

**Fig. 6.**
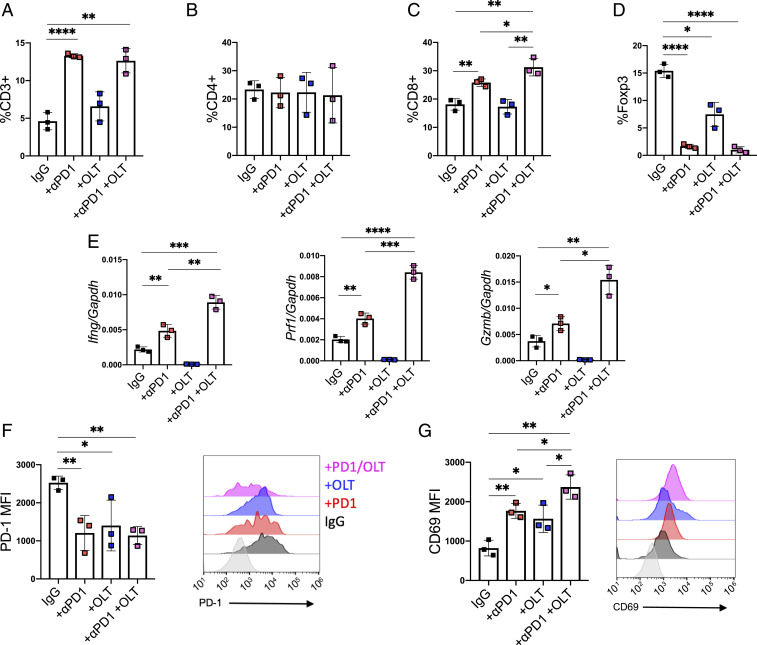
The combination of NLRP3 inhibition and immunotherapy increases the antitumor response compared to the monotherapies. (*A*) Flow cytometry analysis of CD3^+^ T cells present in TME. (*B*) Flow cytometry analysis of CD4^+^ T cells present in the TME. (*C*) Flow cytometry analysis of CD8^+^ T cells present in the TME. (*D*) Flow cytometry analysis of Foxp3^+^ T cells in the TME. (*E*) Gene expression of *Ifng*, *Prf1*, and *Gzmb* in CD8^+^ T cells isolated from primary tumors. (*F*) MFI of PD-1 (*Left*) from CD8^+^ T cells from primary tumors. (*Right*) Representative flow histograms for PD-1. (*G*) MFI of CD69 (*Left*) from CD8^+^ T cells from primary tumors. (*Right*) Representative flow histograms for CD69. Data expressed as mean ± SEM, ****P < 0.0001, ***P < 0.001, **P < 0.01, *P < 0.05.

To better understand the dynamics between MDSCs and T cells in the TME, we performed an in-depth analysis of T cell populations in primary tumors from mice, as described in Fig. 5. The gating strategy for CD3^+^, CD4^+^, CD8^+^, and Foxp3^+^ T cells is shown in *SI Appendix*, Fig. S5*A*. As depicted in Fig. 6*A*, treatment with anti–PD-1, but not OLT1177, significantly increased the percentage of CD3^+^ T cells in the TME compared to mice treated with the IgG control. No changes between the control group were observed in infiltrating CD4^+^ T cells among any of the experimental groups (Fig. 6*B*). Expectedly, anti–PD-1 significantly increased CD8^+^ T cells compared to the control IgG (Fig. 6*C*). Although there was no increase in CD8^+^ cells observed with OLT1177 only (Fig. 6*C*), the combination of anti–PD-1 plus OLT1177 significantly increased the levels of CD8^+^ T cells in the TME when compared to the control IgG (*P* < 0.01) or to the monotherapies (*P* < 0.05 vs. anti–PD-1 and *P* < 0.01 vs. OLT1177) (Fig. 6*C*). Furthermore, a significant reduction (*P* < 0.05) in infiltrating Foxp3^+^ cells was observed with OLT1177. As expected, anti–PD-1 alone as well as the combination of the two inhibitors markedly reduced (*P* < 0.0001) Foxp3^+^ cells in the tumors, compared to the control group (Fig. 6*D* and *SI Appendix*, Fig. S5 *B* and *C*).

We isolated CD8^+^ T cells from the tumors and assessed gene expression of interferon-γ (*Ifng*), perforin (*Prf1*), and granzyme B (*Gzmb*). As shown in Fig. 6*E*, anti–PD-1 treatment increased the expression of *Ifng* compared to the control IgG (*P* < 0.01); however, there was no increase with OLT1177 only treatment. The combination of the two inhibitors resulted in twofold increase (*P* < 0.01) in *Ifng* expression compared to anti–PD-1 only (Fig. 6*E*). *Prf1* levels also showed a significant increase following anti–PD-1 treatment (*P* < 0.01), with no increase with OLT1177 only, but a further twofold increase with the combination treatment (*P* < 0.001) (Fig. 6*E*). A similar increase was also observed for *Gzmb* with anti–PD-1 (*P* < 0.05) but not with OLT1177 only. Again, the combination of these inhibitors increased *Gzmb* gene expression twofold further (*P* < 0.05) (Fig. 6*E*) when compared to the anti–PD-1 only. A pattern emerges from these studies: Anti–PD-1 consistently increases the expression of these genes, NLRP3 inhibition does not, but adding NLRP3 inhibition to anti–PD-1 augments the expression. These data on gene expression in the tumor are consistent with the antitumor outcome of the combination in the mice.

We next assessed PD-1 levels on the surface CD8^+^ T cells using mean fluorescent intensity (MFI). Expression of PD-1 was significantly decreased compared to the control group for each of the experimental groups (*P* < 0.01 vs. anti–PD-1; *P* < 0.05 vs. OLT1177 and *P* < 0.01 vs. PD-1 plus OLT1177) (Fig. 6*F*). We also observed that the early activation marker CD69 was significantly increased in each of the experimental groups when compared to IgG (*P* < 0.01 vs. anti–PD-1; *P* < 0.05 vs. OLT1177 and *P* < 0.01 vs. PD-1 plus OLT1177) (Fig. 6*G*). Notably, the combination of OLT1177 plus anti–PD-1 further increased CD69 expression compared to either monotherapy (*P* < 0.05 vs. both) (Fig. 6*G*).

Next, we analyzed whether the additive effect seen with NLRP3 inhibition on tumor growth was T cell-mediated or a direct effect on tumor cell proliferation. In vitro, B16F10 showed no changes in proliferation when cultured in presence of either 1 or 10 µM OLT1177 compared to the cells cultured in absence of the inhibitor (*SI Appendix*, Fig. S6*A*). In vivo, NCG mice were implanted with B16F10 and fed standard or OLT1177-enriched diet, as described above. As shown in *SI Appendix*, Fig. S6*B*, there was no difference in tumor growth between NCG mice fed standard or OLT1177 diets. Nevertheless, OLT1177-treated NCG mice exhibited a significant reduction in infiltrating MDSCs (both PMN-MDSCs and M-MDSCs) (*SI Appendix*, Fig. S6*C*), consistent with that observed in the wild-type mice (Fig. 5*G*). Overall, these observations indicate that the reduction in tumor growth following NLRP3 inhibition is not mediated by a reduction in tumor cell proliferation or by a direct effect on CD8^+^ T cells, but rather by the reduction MDSCs expansion and their immunosuppressive activities.

## Discussion

Constitutive NLRP3 expression exists in several melanoma cells lines (6), and NLRP3 polymorphisms increase the risk to develop melanoma (10). However, participation of NLRP3 activation in melanoma is not well characterized. In this study, we demonstrate that activation of NLRP3 and the subsequent formation of the NLRP3 inflammasome have clear implications for melanoma progression, mostly by generating a tumor-permissive environment via MDSC expansion. Using cancer databases, we found higher expression of NLRP3 in skin biopsies of melanoma lesions compared to normal skin and with NLRP3 expression positively correlated with expression of IL-1β. Notably, in normal tissues NLRP3 is absent or at very low abundance (28). We demonstrate active NLRP3 inflammasome in malignant melanoma skin biopsies. These observations are consistent with the ability of melanoma cells to exploit an NLRP3 phenotype to gain an advantage over the host. This was particularly apparent when NLRP3 had been specifically inactivated; implanted NLRP3-deficient B16F10 cells resulted in a highly significant reduction in tumors when compared to B16F10 expressing NLRP3 (Fig. 2*A*). The concept of NLRP3 being an intrinsic pathway that participates in melanoma progression has been independently validated by a comprehensive study that relates NLRP3 expression in melanoma cells in response to immunotherapy (29). Rather than a response mechanism to inhibition of checkpoints, the present study revealed that NLRP3 drives melanoma progression independently of checkpoint inhibition.

Expansion of MDSCs often takes place in chronic inflammation (26), yet MDSCs also play a major role in the immunosuppression of cancer (30, 31). In melanoma patients, high levels of MDSCs (both PMN- and M-MDSCs) correlate with stage, metastasis and poor outcomes compared to subjects with lower numbers of these cells (32). High levels of MDSCs in patients with nonresectable melanoma negatively correlate with the clinical response to ipilimumab (anti–CTLA-4) (33–35), and predicts failure of second-line immunotherapy with anti–PD-1 (nivolumab) (36). In subjects with melanoma, inflammatory cytokines tightly associated with IL-1 activity such as IL-6 and IL-8 (20, 37) correlate with MDSCs accumulation and poor clinical outcomes (21). Consistently, we observed in mice that IL-1β inhibition via NLRP3 targeting limited IL-6 levels and the expansion and infiltration of MDSCs in the TME.

The immunosuppressive mechanisms of MDSCs include high expression of PD-L1, IL-10, inducible nitric oxide synthase, TGF-β, and arginase (38–40). Because of the high expression of PD-L1 by MDSCs and the observation of the reduced expansion of MDSCs with OLT1177, we hypothesized that adding OLT1177 treatment to anti–PD-1 would augment the efficacy of this checkpoint inhibitor. We observed that administration of OLT1177 3 d prior to anti–PD-1 treatment significantly reduced tumor volume compared to anti–PD-1 monotherapy. Furthermore, we show that the reduced MDSCs in the TME following preloading of OLT1177 prior to anti–PD-1 significantly increases the percentage of activated CD8^+^ T cells in the TME compared to anti–PD-1 therapy alone. In line with these data, Theivanthiran et al. (29) recently reported that genetic and pharmacological inhibition of NLRP3 reduced the abundance of PMN-MDSCs in the tumor and enhanced the efficacy of immunotherapy. We observed that OLT1177 alone did not change activation of cytotoxic T cells (Fig. 6), and that in NCG mice, we observed no difference in tumor growth between vehicle and OLT1177 treatment. However, in the same mice, the reduction in MDSCs was present, as other tumor-infiltrating lymphocytes may play a role. This hypothesis is supported by the measurable increase of CD69^+^ cells in the TME with OLT1177 treatment. Overall, these data demonstrate that NLRP3 inhibition does not directly influence T cell activity but rather reduces the immunosuppressive environment via MDSCs expansion.

We examined three published clinical studies that employ anti–PD-1 treatment in patients with melanoma (41–43). The analysis revealed that expression of baseline NLRP3/IL-1β levels do not consistently correlate with the IFN-γ signature, which have been positively linked to anti–PD-1 efficacy in melanoma patients. Nevertheless, analysis from the Amato et al. (43) study (*SI Appendix*, Fig. S7) shows a trend of increased expression of NLRP3 and IL-1β in the nonresponders, suggesting a dysregulation of IL-1β and NLRP3 in these patients. Considering the limited number of subjects in these studies, however, further investigations are required in order to establish the exact (positive or negative) role of NLRP3 in immunotherapy response.

Analysis of the TME also revealed a reduction in Foxp3^+^ cells with NLRP3 inhibition (Fig. 6*D*). In humans, Treg levels are increased in several cancers, including melanoma (44) and have been correlated with poor prognosis. Although the mechanism of influence of NLRP3 on Treg activity was not investigated in this study, these data support the hypothesis of NLRP3 as an intrinsic melanoma mechanism that drives inflammation and immunosuppression.

In this investigation we demonstrate the tumor-promoting role for NLRP3 signaling in melanoma. Other studies in a mouse model of colorectal cancer suggest that activation of NLRP3 results in increased antitumor immunity (45, 46), and in a thymoma model NLRP3 activation increased activation of CD8^+^ cells during chemotherapy (47). These seemingly contradictory functions of the NLRP3 inflammasome in tumors are likely time, context, tissue, and cancer specific. In fact, NLRP3 has been shown to be differently regulated in several tumors from leukemia (increased), thyroid carcinoma (increased), lymphoid neoplasm diffuse large B cell Lymphoma (decreased), thymoma (decreased), and uterine carcinosarcoma (no changes), to mention a few. However, consistent with the present data, loss of NLRP3 in melanoma increased antitumoral response and reduced metastasis (29, 48–50). In the worldwide study, the Canakinumab Anti-inflammatory Thrombosis Outcomes Study (CANTOS), over 10,000 patients without cancer were randomized to placebo or a neutralizing antibody to IL-1β for treatment of heart disease (51). After 4 y, there was a significant reduction in heart disease but also a significantly lower incidence in all cancers (*P* = 0.0007) and a 77% highly significant reduction in deaths from lung cancer compared to placebo-treated patients (52). Thus, the neutralization of IL-1β and the inhibition of NLRP3 as shown in this study share the ability to reduce the activities of IL-1β. In conclusion, inhibition of NLRP3 represents an attractive treatment option for inflammatory tumors producing IL-1β and in immunotherapy resistance.

## Materials and Methods

### Cell Culture.

The 1205Lu, A375 and HS294T human melanoma cells were cultured in RPMI, supplemented with 10% FBS, 100 units/mL penicillin, 0.1 mg/mL streptomycin. Cells were maintained in a humidified 5% CO_2_ atmosphere at 37 °C. The cells were plated at 2.5 × 10^5^ per well in a 24-well plate and allowed to adhere overnight. The following day, the media were replaced with fresh RPMI, 10% FBS in the presence and absence of the NLRP3 inhibitor OLT1177 (14). In a separate set of experiments, IL-1β release was induced with recombinant IL-1α (20 ng/mL; PeproTech). In these experiments, OLT1177 was added 30 min prior to stimulation. Supernatants were collected after 24 h. The murine melanoma cells B16F10 and YUMM cells were cultured in DMEM GlutaMax and RPMI, respectively, supplemented with 10% FBS, 100 units/mL penicillin, 0.1 mg/mL streptomycin. B16F10 NLRP3 knockout cells (B16F10 *nlrp3*^*−/−*^; Synthego) were genetically edited by CRISPR-Cas9 technology using the following guide RNA sequence: UUC​CUC​UAU​GGU​AUG​CCA​GG. Forty-eight hours posttransfection, editing efficiency was determined at 96% compared to the control samples using the following PCR and sequencing primers: F:TTT​CCT​GCC​TCC​ATC​TCC​CA and R:TTCAGTGAAGGCGGGTTTCC. Spleen and bone marrow cells were collected from mice fed standard or OLT1177 diet (19) and seeded at 1 × 10^6^ cells per well in a 96-well flat-bottom plate. Supernatants were collected following 48 h for the determination of spontaneous cytokines productions.

### siRNA Transfection.

For siRNA transfection, 1205Lu, A357, and HS294T cells (2 × 10^5^) were incubated with scrambled or NLRP3 siRNA (Santa Cruz Biotechnology). Transfection of the siRNA duplexes (2 nM) was carried out using siRNA Transfection Medium according to the manufacturer’s instructions. After 24 h, the medium was replaced with RPMI 10% FBS (500 µL), and the cells were incubated for an additional 24 h. The supernatants were collected for the measurement of IL-1β levels. Efficacy of the NLRP3 silencing was determined by Western blotting in the cell lysates.

### Cytokine Measurements.

Cytokines levels were measured by specific Qunatikine or DuoSet ELISAs according to the manufacturer’s instructions (R&D Systems).

### Public Database Gene-Expression Analysis.

Normalized gene expression data from the TCGA and the GTEx projects were downloaded from the gene-expression profiling interactive analysis, the TCGA data portals, and Xena.

### Immunofluorescent Microscopy and FRET Analysis.

Human metastatic melanoma biopsies were obtained from US Biomax. Immunofluorescence studies were performed on formalin-fixed paraffin, embedded tissues using a monoclonal antibody to NLRP3 (Cryo-2), a polyclonal antibody to ASC (AL177), or isomolar, species specific, anti-IgG (R&D Systems). Briefly, the sections were deparaffinized, hydrated, and heat-induced antigen retrieval was performed. Next, the sections were placed in a humidified slide chamber and blocked for 1 h in 10% normal donkey serum (Jackson Immunologicals). After removal of the donkey serum, the primary antibodies were added and incubated overnight at 4 °C. The slides were then washed three times with PBS, and cells were incubated for 1 h at room temperature with donkey anti-mouse–Alexa555 (Life Technologies) or donkey anti-rabbit–Alexa488 (Life Technologies) -conjugated secondary antibodies. Nuclei were stained with DAPI (Life Technologies). FRET images were acquired with a Marianas Imaging Station (Intelligent Imaging Innovations) using a Zeiss 639 Plan-Apochromat objective (1.4 N.A.), a Sutter Xenon light source, and a Cooke SensiCam (The Cooke Corporation), as previously described (14). The FRET was calculated as reported previously (14).

### In Vivo Model.

Animal protocols were approved by the University of Colorado Animal Care and Use Committee. Wild-type, *nlrp3*^*−/−*^ (B6.129S6-Nlrp3^tm1Bhk^/J) and NCG (Charles River) mice (8 to 10 wk of age) were purchased from The Jackson Laboratory. B16F10 (2 × 10^5^), B16F10 *nlrp3*^*−/−*^ (2 × 10^5^), and YUMM (5 × 10^5^) cells were mixed with Matrigel (Corning) and then implanted subcutaneously in the hind quarter of mice. Mice were killed after 15 (B16F10) and 18 (YUMM) days from the plug instillation for molecular and cellular analysis. Tumor growth was recorded every 3 d. Mice were fed either an OLT1177-enriched diet or a standard food diet after melanoma cell instillation. Anti-Ly6G (1A8) (8 mg/kg; BioXCell) or the matching IgG (BioXcell) were injected every other day. Anakinra was administrated intraperitoneally daily at 10 mg/kg. Tumor volume was calculated using the formula *V* = (*LW*^2^)/2, where *L* is the length of the longest tumor dimension parallel to the skin containing the tumor midpoint, *W* is the length of the tumor dimension perpendicular to *L* and parallel to the skin, and *V* is tumor volume in cubic millimeters (mm^3^). Dimensions were measured by electronic caliper on restrained mice. Tumors volumes were determined without knowledge of the experimental groups.

### Combination Therapy Model.

B16F10 cells were implanted as described. Four days after implantation, mice were started on the OLT1177 diet or continued on standard diet. At day 7, a neutralizing antibody against PD-1 (8 mg/kg; BioXCell) was injected intraperitoneally every other day. Mice were killed 15 d follow the B16F10 implantation.

Further details of materials and methods are available in *SI Appendix*.

### Statistical Analysis.

Statistical significance of differences was evaluated with a two-tailed Student’s t and log-rank (Mantel–Cox and Gehan–Breslow–Wilcoxon) tests for survival analysis using Prism v7.0 software (GraphPad Software).

## Supplementary Material

Supplementary File

## Data Availability

All study data are included in the article and/or supporting information.
