# Supplementary material for: Targeting tumor-derived NLRP3 reduces melanoma progression by limiting MDSCs expansion
Source: Proc Natl Acad Sci U S A. 2021 Mar 1;118(10):e2000915118. doi: 10.1073/pnas.2000915118 (PMC7958415; doi:10.1073/pnas.2000915118)
Supplement: Supplementary File [file pnas.2000915118.sapp.pdf]

SI Appendix

Targeting tumor-derived NLRP3 reduces melanoma progression by limiting MDSCs expansion

Authors: Isak W. Tengesdal[ab], Dinoop R. Menon[c], Douglas G. Osborne[c],Charles P. Neff[a], Nicholas E. Powers[a], Fabia Gamboni[a], Adolfo G. Mauro[d], Angelo D'Alessandro[e], Davide Stefanoni[e], Morkos A. Henen [ef], Taylor S. Mills[g], Dennis M. De Graaf[ab], Tania Azam[a], Beat Vogeli[e], Brent E. Palmer[a], Eric M. Pietras[g], James DeGregori[d], Aik-Choon Tan[h], Leo A. B. Joosten[b], Mayumi Fujita[c1], Charles A. Dinarello[ab1] and Carlo Marchetti[a1].

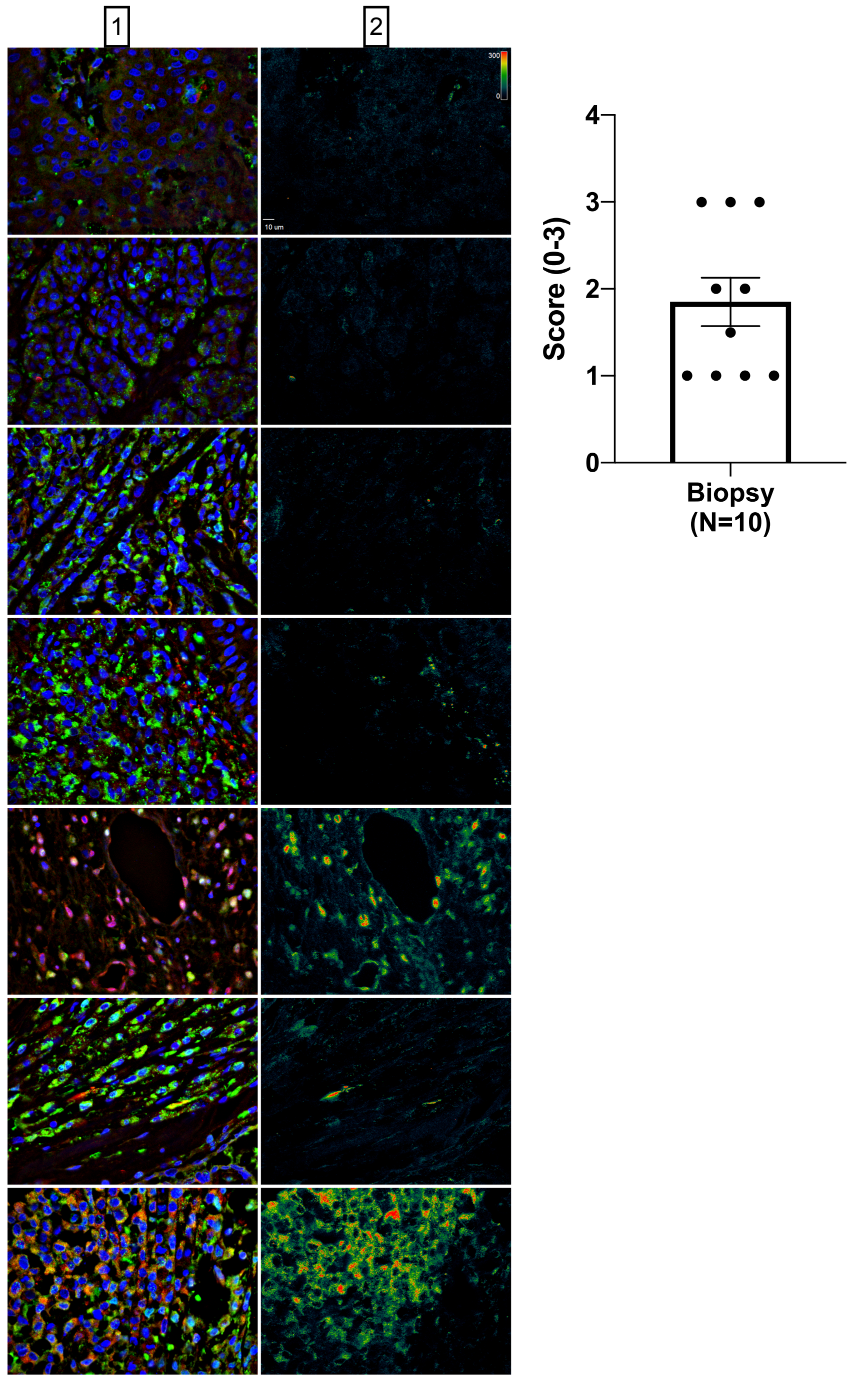

SI Appendix Fig. 1.

Immunofluorescent staining (column 1) and Fluorescence Resonance Energy Transfer (FRET) (column 2) from NLRP3 (red) and ASC (green) in biopsies of human metastatic melanoma (N=7). Quantification of FRET intensity of human metastatic melanoma biopsy from Figure 1d and Supplementary Figure 1 (N=10). The samples were scored macroscopically on a scale from 0 to 3, where 0 = no FRET, 1 =mild FRET, 2 =moderate FRET, and 3 = intense FRET, in increments of 0.5. FRET scoring was performed by two authors.

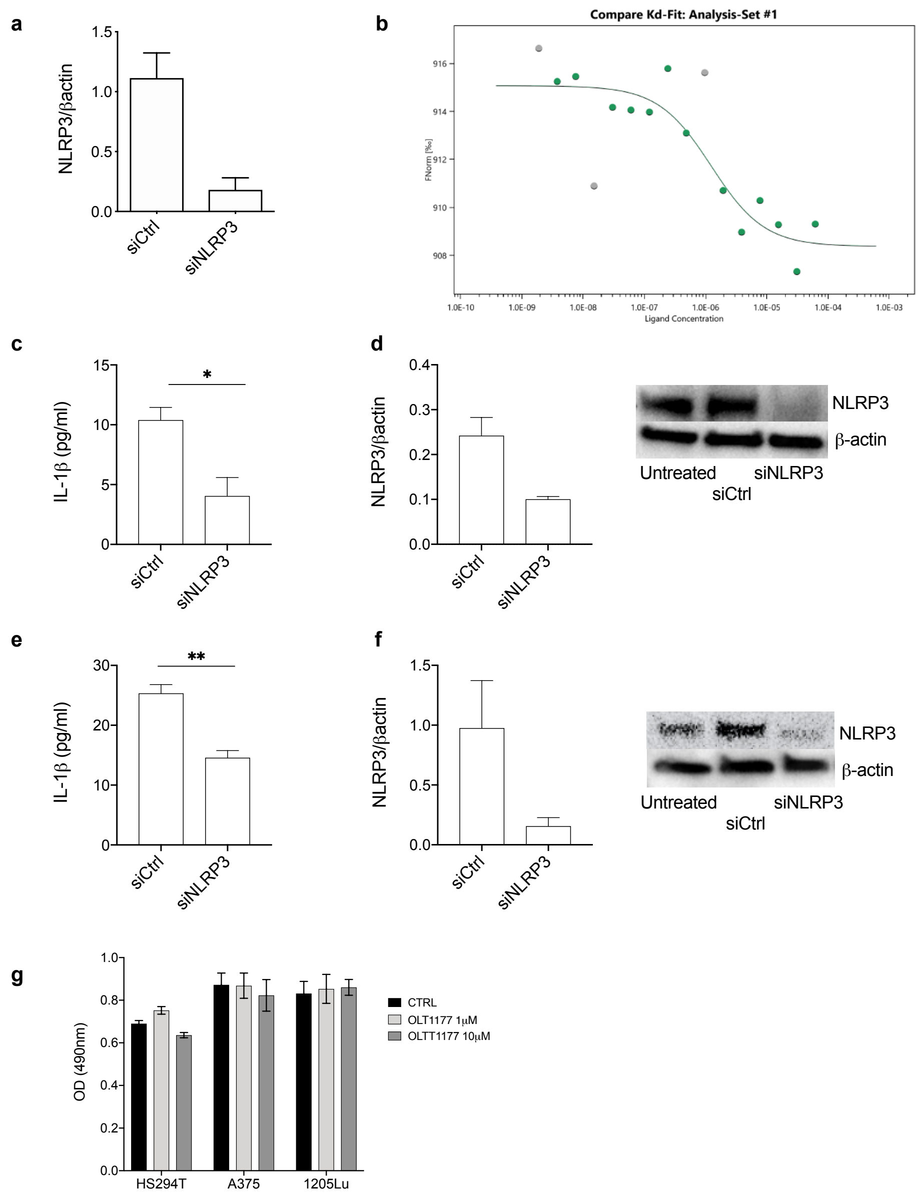

SI Appendix Fig. 2.

(a) Mean ± SEM of NLRP3/β-actin ratio for 1205Lu cells shown in Figure 1f (N=3). **(b)** KD of OLT1177 measured by Microscale thermophoresis (MST). **(c)** Secreted IL-1β levels at 24 hours in A357 cells following transfection with scrambled (siCtrl) or NLRP3 (siNLRP3) small interfering RNA (N=3). **(d)** NLRP3/β-actin ratio (left) for siRNA transfections described in (c) and representative western blot analysis (right) for NLRP3 at 24 hours from A357 cells (Untreated) compared to cells transfected with siCtrl and siNLRP3 (N=3). **(e)** Secreted IL-1β levels at 24 hours in HS294T cells following transfection with scrambled (siCtrl) or NLRP3 (siNLRP3) small interfering RNA (N=3). **(f)** NLRP3/β-actin ratio (left) for siRNA transfections describe in (e) and representative western blot analysis (right) for NLRP3 at 24 hours from HS294T cells (Untreated) compared to cells transfected with siCtrl and siNLRP3 (N=3). **(g)** MTS proliferation assay for HS294T, A357 and 1205Lu cells after 24 hours in presence and absence of OLTT1177 (N=3). $**p<0.01$, $*p<0.05$.

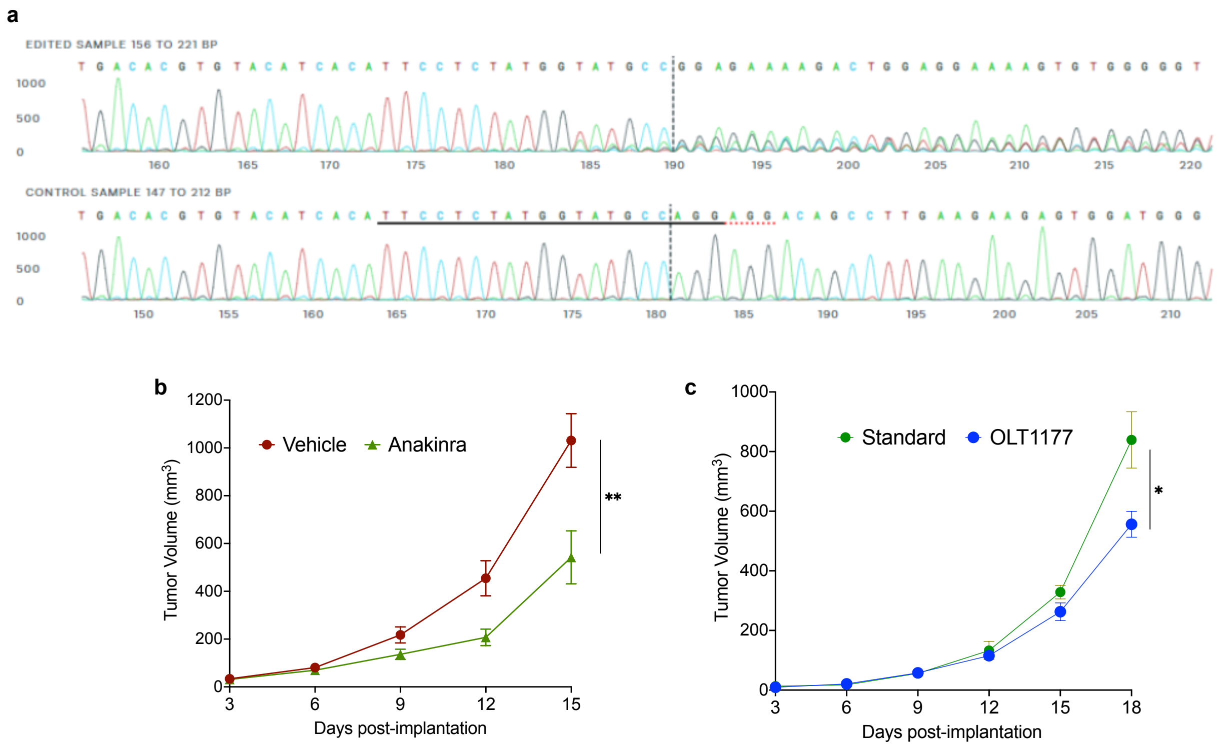

SI Appendix Fig. 3.

(a) Sanger sequence view showing B16F10 CRISPR-Cas9 edited cells (exon 5 specific sgRNA for NLRP3) and non-edited (isogenic negative control; no sgRNA). (**b**)Tumor growth curve (B16F10 cells) of mice treated with anakinra (10mg/Kg daily) (N=10). (**c**) Tumor growth curve (YUMM cells) in wild type mice fed standard or OLT1177 diets (N=10). ***p*<0.01, **p*<0.05.

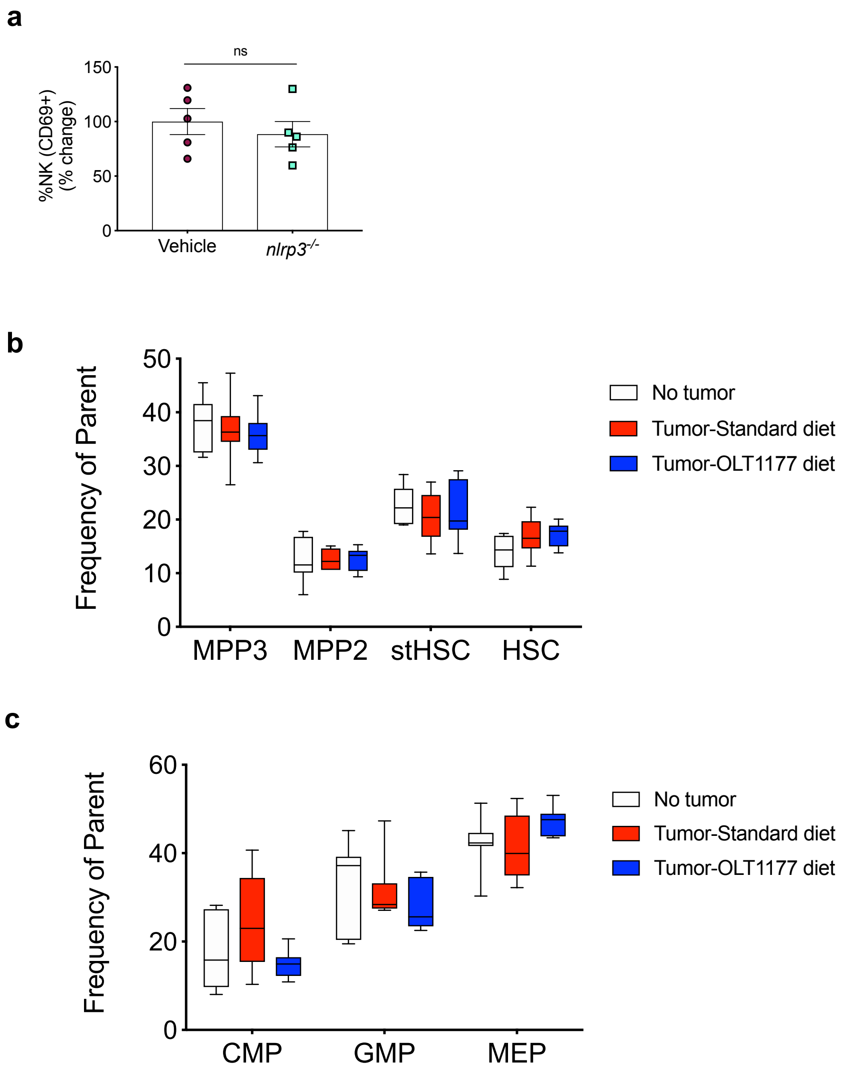

SI Appendix Fig. 4.

(a) CD69+ NK cell levels in primary tumors of WT compared to nlrp3-/- mice (N=5/group).

(b,c) Flow cytometry analysis of hematopoietic stem and progenitor cells (HSPCs) (b) and common myeloid precursor (CMP) (c) cells in bone marrow of non-tumor mice or tumor-bearing mice fed standard or OLT1177 diets (N=7-8/group). MPP3 (multipotent progenitor 3), MPP2 (multipotent progenitor 2), stHSC (short-term hematopoietic stem cell), HSC (hematopoietic stem cell), CMP (common myeloid progenitor), GMP (granulocyte-monocyte progenitor), MEP (megakaryocyte-erythroid progenitor).

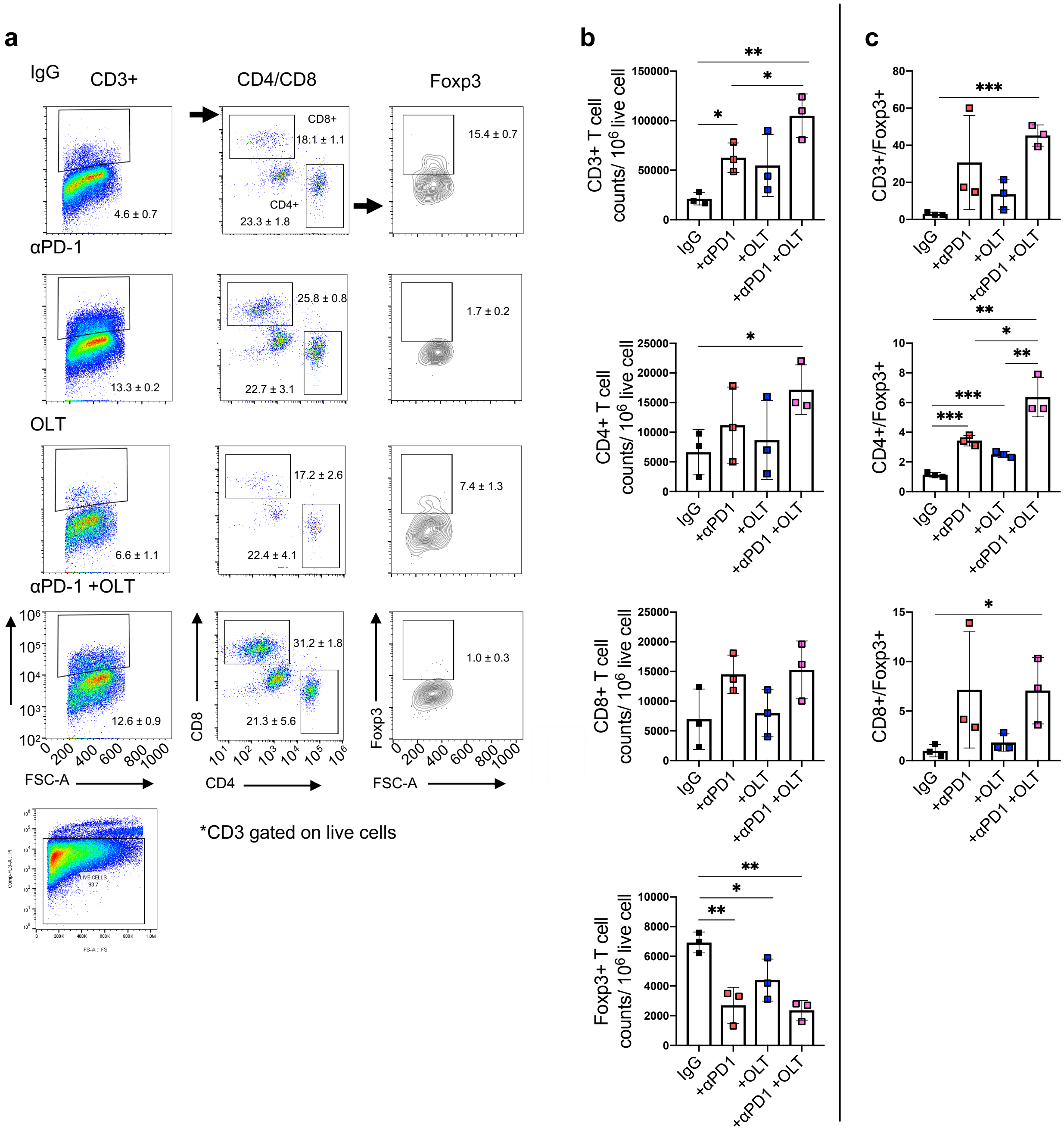

SI Appendix Fig. 5.

(a) Gating strategy for $CD3^+$, $CD4^+/CD8^+$, and $Foxp3^+$ live cells in the TME from control (IgG) mice or mice treated with anti-PD-1, OLT1177 or combination of anti-PD-1 and OLT1177. Prior to CD3 gating, live cell gating was performed using propidium iodide. Arrows indicate direction of gating strategy. **(b)** Quantification of the percent and cell count/10^6 live cells for $CD3^+$ (top), $CD4^+$ (top middle), $CD8^+$ (bottom middle) and $Foxp3^+$ (bottom) T cells present in the TME from (a) (N=3). **(c)** Ratio of $CD3^+$ (top), $CD4^+$ (middle) and $CD8^+$ (bottom) T cell to Foxp3 T cell in the TME from (a) (N=3). ***$p<0.001$, **$p<0.01$, *$p<0.05$.

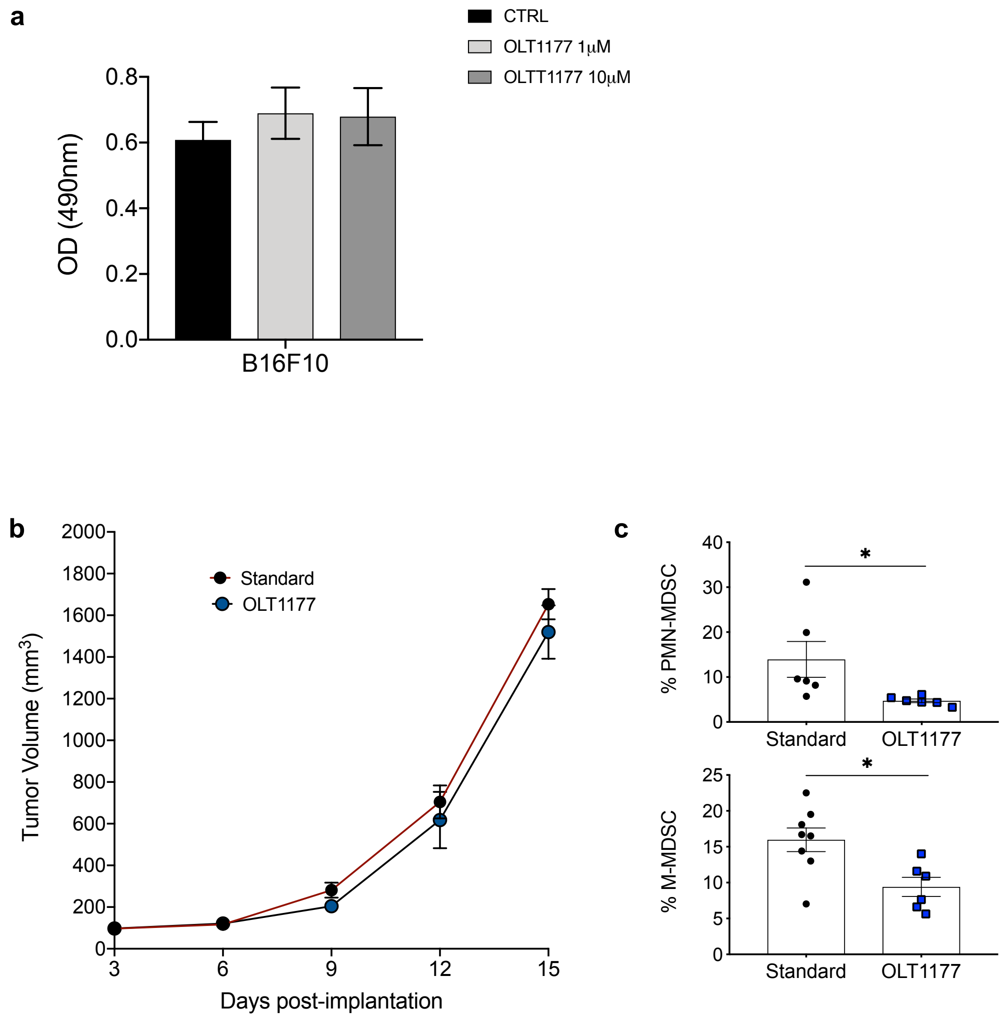

SI Appendix Fig. 6.

(a) MTS proliferation assay for B16F10 cells after 24 hours in presence and absence of OLTT1177 (N=3). **(b)** Tumor growth in NCG mice fed standard or OLT1177 diet (N=8/group). **(c)** PMN-MDSC and M-MDSDC populations present in primary tumors from mice described in (b) (N=6-8). *p<0.05.

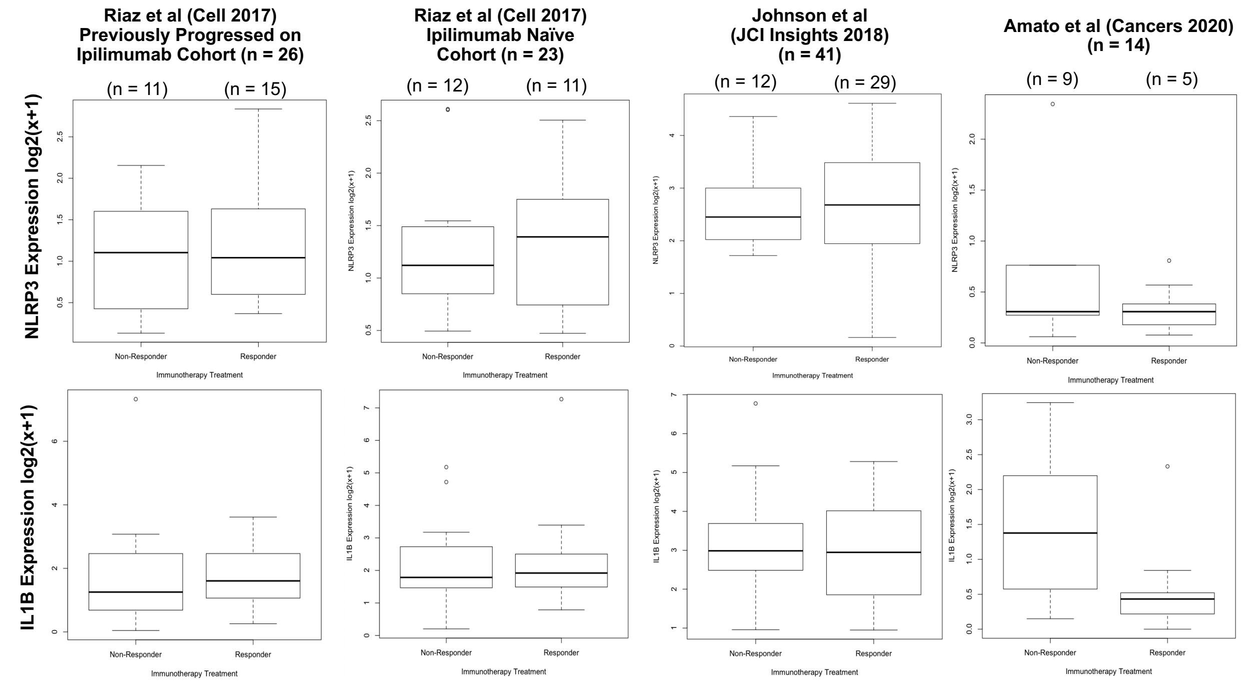

SI Appendix Fig. 7.

Expression of NLRP3 and IL-1β in melanoma patients following immunotherapy. *p>0.05 Mann Whitney U test in all cohorts. References (1)(2)(3).

Methods

Microscale thermophoresis (MST). Thermophoresis measurements were carried out on a NanoTemper Monolith NT.115 pico-instrument (NanoTemper Technologies GmbH) at 25˚C using medium power and 20% excitation power (auto-detect-pico-red). NLRP3 protein (Novus, CO) was fluorescently labeled with 100µl Red-NHS 2nd generation dye (60µM concentration). The reaction mixture was incubated at room temperature for 30 minutes followed by centrifugation for 10 minutes at 4˚C and 15000 g speed. 5 nM of the labeled NLRP3 and series dilution of OLT1177 were loaded into 16 standard capillaries (NanoTemper Technologies GmbH). Data were analyzed using the NanoTemper software (MO affinity analysis v 2.2.7).

MTS cell proliferation assay. Cell proliferation was determined according to manufacturer's recommendations (Abcam). B16F10, HS294T, A357 and 1205 Lu cells were cultured as described above in presence and absence of OLT1177 for 24 hours and absorbance was determined at 490nm.

Western blotting. 1205Lu, A357 and HS294T cells were lysed in RIPA buffer supplemented with protease inhibitors (Roche), centrifuged at 13,000g for 20 min at 4 °C and the supernatants were obtained. Protein concentration was determined in the clarified supernatant using Bio-Rad protein assay (Bio-Rad Laboratories, Hercules, CA). Proteins were electrophoresed on Mini-PROTEAN TGX 4–20% gels (Bio-Rad Laboratories) and transferred to nitrocellulose 0.2µm (GE Water & Process Technologies). Membranes were blocked in 5% dried non-fat milk in PBS-Tween 0.5% for 1 hour at room temperature. Primary antibodies for NLRP3 1:1000 (Cryo-2, AdipoGen San Diego, CA), ASC 1:1000 (AL177, AdipoGen) and IL-1β 1:1000 (R&D System) were used in combination with peroxidase-conjugated secondary antibodies. A primary antibody against β-actin (Santa Cruz Biotechnology) was used to assess protein loading.

Flow Cytometry. Bone marrow, spleen and lymph nodes were harvested and homogenized using 40µm cell strainers. Tissue-derived cells were then washed and resuspended in RPMI at $5x10^6$/ml. Single cells suspension were stained using anti-CD45 Pe/Cy7 (BioLegend, San Diego, CA), anti-CD11b BV785 (BioLegend), anti-CD3 Alexa Fluor700 (eBioscience, San Diego, CA), anti-Ly6G PacBlue (BioLegend), anti-Ly6C PerCp/Cy5.5 (Biolegend), anti-CD-161 APC (BioLegend), anti-CD335 BV650 (BioLegend), anti-CD69 BV421 (BioLegend) and CD8 Alexa Fluor 488 (BioLegend). Hematopoietic cell staining was performed using the definitions: HSC (lin-, Sca1+, cKit+, Flk2-, CD150+, CD48-, ESAM+) stHSC (lin-, Sca1+, cKit+, Flk2-, CD150-, CD48-) MPP2 (lin-, Sca1+, cKit+, Flk2-, CD150+, CD48+) MPP3 (lin-, Sca1+, cKit+, Flk2-, CD150-, CD48+) MEP (lin-, Sca1-, cKit+, FcγR-, CD34-) GMP (lin-, Sca1-, cKit+, FcγR+, CD34+) CMP (lin-, Sca1-, cKit+, FcγR-, CD34+). Anti-lineage cocktail in PE/Cy5: anti-B220 (Biolegend), anti-CD4 (eBiosciene), anti-CD5 (Biolegend), anti-CD8a (Biolegend), anti-Gr1 (Biolegend), anti-CD11b (Biolegend), anti-Ter119 (Biolegend). HSPC cells were stained with anti-Sca1 BV421 (Biolegend), anti-Flk2 Biotinylated (Biolegend), anti-Streptavidin BV605 (Biolegend), anti-CD150 BV785 (Biolegend), anti-CD34 FITC (eBioscience), anti-FcγR PerCP-

eFluor710 (eBioscience), anti-ESAM APC (Biolegend), anti-CD48 Alexa fluor700 (Biolegend), anti-cKit (CD117) APC/Cy7 (Biolegend).
Tumors were digested as described elsewhere (PMID: 27325269). Single cell suspensions from the tumors were Fc blocked with purified anti-mouse CD16/CD32 monoclonal antibody (2.4G2) followed by staining for T lymphocytes using stains for live cells, CD3, CD8, CD4, CD69, PD-1, and Foxp3 using the Foxp3 Cytoperm/Cytofix staining kit (BD Pharmingen, San Diego, CA) or for analyzing monocyte population using stains for live cells, CD45, CD11b, Ly6C and Ly6G. Lymphocyte gating strategy shown in Fig. S5a and 6c.

T cell isolation

CD8+ T cells were purified from tumors of either control (IgG), +OLT1177, +PD-1, and +OLT177/PD-1 mice using the mouse CD8+ T cell Isolation Kit (Miltenyi Biotec). Total RNA was extracted from the purified lymphocytes using the RNeasy Plus Mini Kit (Qiagen) and subsequently reverse-transcribed using the iScript cDNA Synthesis Kit (Bio-Rad). Quantitative RT-PCR (qRT-PCR) was performed with Power Up SYBR Green PCR Master Mix (Applied Biosystems, Foster City, CA) on the AriaMx Real-Time PCR system (Agilent Technologies, Santa Clara, CA). Primer sets used for mouse cells were the following: GAPDH forward—5′C ACC ACC AAC TGC TTA GC-3′; reverse—5′-GGC ATG GAC TGT GGT CAT GAG-3′; IFN-γ forward—5′-CCA GGA CAG ACC ACA TTC A-3′; reverse—5′-CTG GAC ACC CAT TCC AGA CT-3′; perforin forward—5′-CCT GTG AGG AGA AGA AGA AG-3′; reverse—5′-TCG TTA ATG GAG GTG TGA TGG-3′; granzyme b forward—5′-CTT CCT GAT ACG AGA CGA CTT C-3′; reverse—5′-CGG CTC CTG TTC TTT GAT ATT G-3′;. Quantification of mRNA was measured using change-in-cycling-threshold (ΔCt) and is shown relative to GAPDH mRNA expression.

References

1. Riaz N, *et al*. (2017) Tumor and Microenvironment Evolution during Immunotherapy with Nivolumab. *Cell* 171(4):934-949 e916.
2. Johnson DB, *et al*. (2018) Tumor-specific MHC-II expression drives a unique pattern of resistance to immunotherapy via LAG-3/FCRL6 engagement. *JCI Insight* 3(24).
3. Amato CM, *et al*. (2020) Pre-Treatment Mutational and Transcriptomic Landscape of Responding Metastatic Melanoma Patients to Anti-PD1 Immunotherapy. *Cancers (Basel)* 12(7).
